# On the Implications of a Sex Difference in the Reaction Times of Sprinters at the Beijing Olympics

**DOI:** 10.1371/journal.pone.0026141

**Published:** 2011-10-19

**Authors:** David B. Lipps, Andrzej T. Galecki, James A. Ashton-Miller

**Affiliations:** 1 Department of Biomedical Engineering, University of Michigan, Ann Arbor, Michigan, United States of America; 2 Department of Biostatistics, School of Public Health, University of Michigan, Ann Arbor, Michigan, United States of America; 3 Institute of Gerontology, University of Michigan, Ann Arbor, Michigan, United States of America; 4 Department of Mechanical Engineering, University of Michigan, Ann Arbor, Michigan, United States of America; 5 School of Kinesiology, University of Michigan, Ann Arbor, Michigan, United States of America; Federal University of São Paulo, United States of America

## Abstract

Elite sprinters offer insights into the fastest whole body auditory reaction times. When, however, is a reaction so fast that it represents a false start? Currently, a false start is awarded if an athlete increases the force on their starting block above a given threshold before 100 ms has elapsed after the starting gun. To test the hypothesis that the fastest valid reaction times of sprinters really is 100 ms and that no sex difference exists in that time, we analyzed the fastest reaction times achieved by each of the 425 male and female sprinters who competed at the 2008 Beijing Olympics. After power transformation of the skewed data, a fixed effects ANOVA was used to analyze the effects of sex, race, round and lane position. The lower bounds of the 95, 99 and 99.9% confidence intervals were then calculated and back transformed. The mean fastest reaction time recorded by men was significantly faster than women (p<0.001). At the 99.9% confidence level, neither men nor women can react in 100 ms, but they can react in as little as 109 ms and 121 ms, respectively. However, that sex difference in reaction time is likely an artifact caused by using the same force threshold in women as men, and it permits a woman to false start by up to 21 ms without penalty. We estimate that female sprinters would have similar reaction times to male sprinters if the force threshold used at Beijing was lowered by 22% in order to account for their lesser muscle strength.

## Introduction

Sprinters competing in the Olympic Games arguably achieve some of the fastest possible human auditory reaction times involving whole body responses. After years of training, they are highly motivated to leave the starting blocks quickly since medals can be decided by a few milliseconds. For example, Leroy Burrell defeated Carl Lewis by 30 ms in the 100 m sprint at the 1992 Barcelona Olympic Games primarily due to his 49 ms faster reaction time at the start. Therefore, the first question we address in this paper concerns the fastest possible whole body auditory reaction time in highly trained and motivated Olympic sprinters.

Women not participating in the Olympic Games have been reported to exhibit slower simple reaction times than men [1]. This raises the possibility of a sex difference in the fastest reaction times recorded by elite sprinters at the Olympic Games. In a study of the effect of lane position on sprinter reaction time, female sprinters at the 2004 Athens Olympics did exhibit slower mean reaction times than male sprinters [2]. Despite analyses of reaction times at international sprinting competitions [2], [3], none have examined whether a sex difference exists in the fastest possible reaction times of sprinters. Using data made publicly available on the 2008 Beijing Olympics web site, we examined the second question in this paper, namely, that a sex difference exists in the fastest sprinter reaction times.

A related question concerns the method for determining whether a highly-trained athlete has reacted so fast that (s)he must have false started (for example, [4]–[6]). This was keenly debated, for example, after Linford Christie was disqualified from the 100 m finals of the 1996 Atlanta Olympic Games after two false starts, the year that both he and his arch rival, Michael Johnson, arrived at the Olympics having broken the 100 m world record. The International Association of Athletics Federation (IAAF) rule 161.2 stipulates that a false start occurs when a sprinter when a reaction time “is less than 100/1000ths of a second” [7]. In practice the method used to detect a false start varies with the company awarded the timing contract for the competition [5], [8]. At the Beijing 2008 Olympic Games Swiss Timing, Ltd (Corgemont, Switzerland) performed the false start detection and sprint race timing using Omega equipment. In past Olympics, a false start was considered to occur when the increase in force applied by the sprinter to the starting blocks exceeded a given increase in force (i.e. ‘threshold’) before 100 ms has elapsed from the start gun [6], [8]. Part of the IAAF justification for the 100 ms criterion was a study on the auditory reaction times of eight Finnish sprinters [4]. However, none of those participants were Olympic-level athletes, none were actually competing in the Olympic Games, and no women were included. This leaves open a third question that will be addressed in this paper, namely the force threshold used for the false start criterion in men and women. To address this we will test the hypotheses that the minimum valid sprinter reaction time in men and women is 100 ms.

## Methods

The reaction time data publicly available on the 2008 Beijing Olympics website (http://en.beijing2008.cn/) included round data from the heats, quarterfinals, semifinals, and finals for the 100 m, 200 m, 400 m sprints, along with the 110/100 m hurdles. Data were collected for 224 male sprinters (439 total male races) and 201 female sprinters (387 total female races), who participated in up to eight races. All names were stripped from the record to blind the analyses. Each reported reaction time was the elapsed time in ms from the onset of the gun signal to the instant that the force applied by the athlete to the start block(s) increased to a specified force threshold set by Swiss Timing Ltd. (Corgemont, Switzerland). (Responding in July, 2011 to an email requesting the value of the force threshold(s) used for sprinters at the Beijing Olympic Games, a representative of Swiss Timing replied ‘Unfortunately this kind of information is not public’.) The fastest valid reaction time was selected for each sprinter in order to assume independence within the data set. The reaction time values for the 25 men and 4 women who false started were not specified. In this study, we retain the term ‘reaction time’ that is used in international athletic competitions when evaluating whether a false start has occurred. However, in the scientific literature, this term would be termed the ‘response time’, which is the sum of the premotor time, the electromechanical delay, and the time required to generate a given foot force against the starting block.

Statistical analysis was performed using PASW Statistics 18 (SPSS Inc., Chicago, IL). Previous investigations of international sprinting competitions fail to acknowledge the lack of normality within the data set [2], [3]. Since the data are right-skewed, the data were power transformed (−1.5 power) and normality was confirmed with the Shapiro-Wilk test. A fixed effects ANOVA was used to analyze the effect of sex, race, round, and lane position on the transformed reaction time data. The median and 95% confidence intervals were calculated for the sex, race, and round factors, and contrast estimates were used to compare within each ANOVA factor. For the sex factor, we calculated the lower bounds of 95^th^, 99^th^ and 99.9^th^ % confidence intervals, which provide insight into the best possible reaction times for the men and women, respectively. The upper bounds of the confidence intervals are not relevant to the question at hand. The mean, median and confidence intervals for the men and women reaction times were then back-transformed to the original temporal scale.

## Results

**Figure 1:**
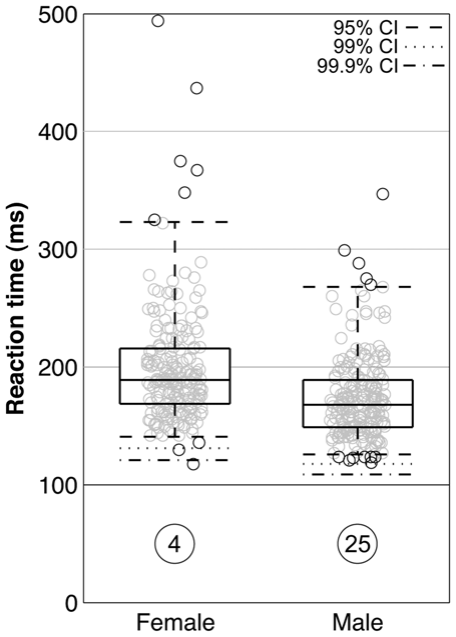
Sex difference in sprinter reaction times. Scatter and box-whisker (including median, quartiles, and 95% confidence interval [CI]) plots of the fastest valid reaction times of sprinters at the Beijing Olympics. The lower bounds of the 99% and 99.9% CI are also shown. The numbers of false starts are circled.

The mean fastest reaction times were 23 ms shorter in men than women (166 ms vs 189 ms, respectively; F(1,409) = 108.846; p<0.001; Fig. 1). The lower bounds of the 99% confidence intervals were 118 ms for men and 131 ms for women. The lower bounds of the 99.9% confidence interval show the fastest possible male sprinter reaction time to be 109 ms, and the fastest female reaction time to be 121 ms. We therefore rejected the hypothesis that the fastest possible reaction time is 100 ms for the particular force threshold(s) used. This conclusion is supported by the absence of any reaction times between 100 ms and 117 ms, and the fact that 14 individuals (12 men) had times between 118 ms and 130 ms. Both results suggest that the reaction times below 100 ms were correctly classified as false starts.

Race (F(3,409) = 85.208; p<0.001) and round (F(3,409) = 23.004; p<0.001) were also significant factors, while lane position (F(8,409) = 0.991, p = 0.442) was not significant. Athletes in the shorter sprint races, specifically the 110/100 m hurdles, exhibited the fastest reaction times (Tables 1–2). The semifinals and preliminary heats produced significantly faster reaction times than the final round (Table 2), but we note the smaller sample sizes for those heats (Table 1).

**Table 1:** Descriptive statistics for Olympic sprinter reaction times (ms).

Factor		N	Median	95% CI
Sex	Male	224	168.0	(126.4–267.9)
	Female	201	189.0	(140.5–322.5)
Race	100	157	170.0	(129.6–255.5)
	110H	79	161.0	(125.8–229.7)
	200	87	178.0	(137.9–268.6)
	400	102	209.0	(152.1–402.3)
Round	Finals	180	177.5	(135.3–272.0)
	Semifinals	59	169.0	(123.7–310.2)
	Quarterfinals	173	179.0	(130.7–339.8)
	Preliminary Heats	13	181.0	(122.5–282.7)

N - sample size; CI - confidence interval; H – hurdles.

**Table 2:** Contrast estimates for transformed Olympic sprinter reaction times.

Factor	Contrast	Estimate	SE	p-value
Sex	Female	−1.496	0.143	<0.001
	(vs. Male)	0		
Race	110H	2.169	0.274	<0.001
	200	0.157	0.273	0.566
	400	−4.140	0.282	<0.001
	(vs. 100)	0		
Round	Semifinals	1.107	0.358	0.002
	Quarterfinals	−0.138	0.291	0.636
	Preliminary Heats	1.307	0.614	0.034
	(vs. Finals)	0		

SE – standard error. Contrast estimates are based on transformed data (−1.5 power). Negative contrast estimates indicate slower reaction times (ms).

## Discussion

This paper is novel in its focus on the fastest reaction time achieved by each sprinter, irrespective of race or round. Given a 99.9% confidence interval, for the particular force threshold(s) used, it is unlikely that any human can react in less than 100 ms unless they anticipate the start gun. But a lower starting block force threshold would systematically result in faster measured reaction times, as discussed below. Shorter distance sprints did result in faster starting block reaction times, confirming results from international athletic competitions [3]. While a sprinter's reaction time has been reported to be shortest when competing in the final round [3], our results showed similar reaction times across round, given the uneven sample sizes present when considering the effect of round (Table 1). This study did not corroborate the effect of lane position on the fastest reaction times of sprinters previously seen at the 2004 Athens Olympics [2], likely because the 2008 Beijing Olympics were the first to use speakers behind each starting block to amplify the starter's gun.

### Sex Differences in Reaction Times

The sex difference in the mean fastest reaction time corroborates and extends previous results from the 2004 Athens Olympics that showed male sprinters (163 ms) averaged significantly faster reaction times than female sprinters (188 ms) [2]. While the present study purposely used the fastest time achieved by each athlete, the study of the Athens Olympics data was mainly focused on the effect of lane position and included multiple data points, if available, from each individual. This means that the previous study had a lack of data independence when considering the effect of sex.

Since women have faster auditory latencies [9], [10] and shorter neural pathways due to a shorter stature than men [11], the origin of the slower female Olympic reaction times would appear to be peripheral and not central. Indeed, the plantarflexor premotor time in response is significantly shorter in healthy young women [12]. However, healthy young women do have a 20% lower rate of developing plantarflexor strength and 28% lower maximum isometric strength than men [12], presumably due to their 32% smaller leg muscle mass [13]. This peripheral motor factor will have systematically lengthened the time the women sprinters required to increase their force to the specified threshold on the 2008 Beijing Olympic starting blocks.

### Implications for Detecting a False Start

Our results suggest that an athlete who manages to react by reaching the threshold force on the starting blocks between the 100 ms criterion used at the Beijing Olympics and the lower limit of the present 99.9% confidence interval must have anticipated the gun and thereby gains an unfair advantage over other competitors in that race; this is especially true for the women in that their ‘window of opportunity’, 21 ms (calculated as 121 ms–100 ms), is twice as long as that of the men. In order to provide equal opportunity within sex, the use of sex-specific start criteria appears warranted. In our search of the literature and the internet, we could find no suggestion that sex-specific force thresholds are currently being used in athletic sprint competitions.

Starting block reaction times are sensitive to the force threshold selected by the timing company. Swiss Timing holds the value of the force threshold(s) used at the 2008 Beijing Olympics as proprietary information (see Methods), and no specifics regarding the exact mechanism of detecting a false start is provided in the patent filed by Omega [14]. But a 25 Kgf threshold for both males and females has become a de facto standard (for example, [6], [15]), so in the discussion that follows we shall assume this value was used at the 2008 Beijing Olympics.

**Figure 2:**
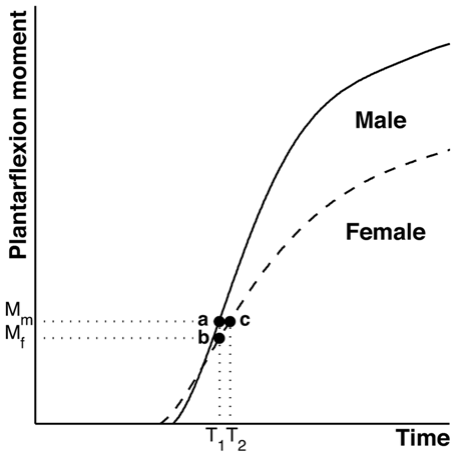
Sex difference in rapid plantarflexion moment development. At time (T_1_) the moments produced by men (M_m_) and women (M_f_) are indicated by points *a* and *b*, respectively. The times taken by men (T_1_) and women (T_2_) to develop the plantarflexion moment, M_m_, are indicated by points *a* and *c*, respectively. Figure is redrawn from [9].

The increase in propulsive force on the starting blocks is primarily developed by rapid bilateral increases in ankle plantarflexion moment and secondarily by hip extension moments [4], [16]; neither knee contributes much in this regard [16]. When the plantarflexors contract, the reaction force on the foot from the starting block is approximately proportional to the plantarflexion moment divided by the lever arm of that force about the center of the ankle joint. For a given plantarflexion moment, the force developed by healthy young females will be 11% larger than healthy young males due to their smaller foot lever arms [17] (Text S1). However healthy young men, on average, develop 20% greater plantarflexion moment than young women do in the same amount of time (c.f., points ‘a’ and ‘b’ in Fig. 2, [12]). Therefore, healthy young men would develop an 6% larger force on the starting blocks than would healthy young women in the same time interval, T_1_ (Fig. 2 and Text S1). These calculations would likely change for sprinters given that they are stronger and develop force at a faster rate than healthy young adult non-sprinters [18], [19].

Indeed, male sprinters have 21% greater plantarflexion strength than female sprinters (in addition to 47% greater hip extension strength and 56% greater knee extension strength) [20]. Furthermore, the rate of maximal plantarflexor force development of male sprinters is 25% greater than healthy young males [18], while female sprinters have 7% greater rate of plantarflexor force development than healthy young females [19]. With these facts under consideration, one can calculate that female sprinters should develop 19.4 Kgf in the time it takes male sprinters to generate 25 Kgf on a single block (Text S1). If false start detection systems examine the rate of force increase, rather than using a set force threshold, these would need to take into account the sex difference in the rate of maximal plantarflexor force development, since females sprinters have 22% lower rate of force development in their plantarflexors when compared to male sprinters [18], [19].

Our calculations suggest that the allowable increase in force on the starting block 100 ms after the gun fires should be 19.4 Kgf for women if the male force threshold remains unchanged at 25 Kgf. For the current starting block force threshold of 25 Kgf, we calculate that female sprinters will physiologically require a 7% longer time to reach the same value as men. This motor difference explains over half of the 11% sex difference in the lower bounds of the 99.9% confidence intervals we found. As a side note, from Newton's second law, since female sprinters have 25% less body mass than male sprinters [11] and 22% less propulsive force in the same time as men (see Text S1), women might accomplish 4% greater acceleration than men.

The present results do not support the suggestion to reduce starting block reaction times to as low as 80 ms [2], [3] unless the force threshold(s) used for the Beijing starting blocks are lowered accordingly. The present study identifies a sex difference in the fastest reaction times of Olympic sprinters that has previously been overlooked through the focus on mean times. Although the fastest valid reaction time (118 ms) was achieved by a woman, this value is an outlier at the 99.9% level (Fig. 1). Our analysis suggests this sprinter probably anticipated the start gun since no other female sprinter recorded a reaction time faster than 130 ms, but she was competing fairly under the rules then in effect.

The significance of the present results extends beyond athletic competition. Consider, for example, the emergency use of a brake pedal to arrest the forward momentum of an automobile in order to avoid a collision. One can make the case that the automobile brake control system should be informed whether a male or female is driving the car because, a 6% reduction is needed in a 25 Kgf brake pedal activation force for women to develop equal reaction times to males (Fig. 2 and Text S1).

The strengths of this study include the use of only the fastest reported reaction time for each athlete and the fact that the statistical analysis takes into account the skewed raw data. Another strength from a motor control perspective is the analysis of an overlearned task, so little learning or adaption will occur as the heats progress at an athletic competition. Therefore, it is unlikely that more practice would shorten the fastest reaction time of an Olympic sprinter, but it might decrease their variability.

A potential limitation of the paper is the censoring (truncation) of data points below 100 ms in our statistical analysis. Since the reaction times were measured at the Olympics to determine a false start, no data points below 100 ms could be included in the analysis because they were not reported in the official results. However, the lack of any data between 100 ms and the shortest recorded valid reaction time of 118 ms suggests that data points below 100 ms were correctly identified as false starts. We considered the confidence interval of 99.9% in our analysis because most top-level sprinters undertake 100 starts, but few, if any, undertake 1,000 starts in a year of outdoor competition. Hence, our use of the 99.9% confidence intervals is conservative and would have included all valid reaction times for the best competitors of the 2008 season. We acknowledge that the 100 m races began from a straight start, while the 200 m and 400 m races began with a staggered start. Since the Omega equipment used at the 2008 Beijing Olympics utilized both the start gun and a speaker behind the each lane, it is possible that runners in the staggered lanes closest to the starter might have a slight advantage if they responded to the gun instead of the speaker. However, our ANOVA results suggest that lane position did not have a significant effect on the fastest reaction times of sprinters. Lastly, we cannot exclude the possibility of a ‘guess factor’ in this data set: the new IAAF guidelines, which automatically disqualify a sprinter on the first false start and hence attempt to minimize this factor, were not implemented until after the 2008 Beijing Olympics.

In conclusion, the results lead us to reject the hypothesis that the minimum reaction time of male or female sprinters is 100 ms under the start rules used for the Beijing Olympic sprinters. The slower apparent reaction time of women is caused by requiring the sex with the lower strength to develop the same increase in force as the men in order to determine if a false start occurred. A revision of the Beijing false start force criterion seems warranted in order to yield equal racing opportunity for women, for the reasons above. The most practical solution is combining the current 100 ms IAAF criterion with a 22% lower starting block force threshold for women, while keeping the force threshold for men at the same value as at the Beijing Olympics. We anticipate the number of female false starts would increase to that of the men's sprint races at the 2008 Beijing Olympics, but it would be unlikely to change the overall race time because of the low probability of any athlete being able to develop very fast reaction times.

## Supporting Information

Text S1 Mathematical model to estimate the sex difference in starting block force generation.
(DOCX)
