# Supplementary material for: On the Implications of a Sex Difference in the Reaction Times of Sprinters at the Beijing Olympics
Source: PLoS One. 2011 Oct 19;6(10):e26141. doi: 10.1371/journal.pone.0026141 (PMC3198384; doi:10.1371/journal.pone.0026141)
Supplement: Text S1 — Mathematical model to estimate the sex difference in starting block force generation. (DOCX) [file pone.0026141.s001.docx]

**TEXT S1**

Three methods have been used for defining the allowable increase in force on the starting blocks prior to 100 ms having elapsed from the start gun [1,2]. One method is to define an upper bound on the increase in force (i.e., 25 Kgf [3]) that a sprinter can apply to the starting block within 100 ms. A second is to use a measure of the rate of increase of the force applied to the start block. A third is to use an accelerometer on the start block to detect the onset of forward propulsion by the sprinter. In what follows we consider the first method because of its apparent use at 2008 Beijing Olympics.

It is presently unclear what value of the force threshold should be used to decide whether a female sprinter has false started. Thelen et al. demonstrated that females have a significantly faster premotor time but slower rate of generating plantarflexor force [4]. One approach is to determine the maximum force that a female sprinter can apply to a starting block in the time that a male sprinter takes to develop 25 Kgf on the starting blocks. This value then becomes an initial estimate for the force threshold that would be needed to determine whether a female sprinter has false started at the Olympics.

A first estimate for the female threshold can be made by linearly interpolating the Thelen et al rate of isometric plantarflexion torque data for healthy young adults (Table 1 in [4]). If we multiply the putative male starting block force threshold of 245 N (25 Kgf) by the estimated lever arm that force has about the ankle joint, we can calculate the plantarflexion moment involved. By utilizing foot anthropometric measurements [5], we can estimate the length of a lever arm acting at the first metatarsal joint as being 0.143 m for the male. This would result in a male plantarflexion moment of 35 Nm. From Thelen et al, this moment would take males 238 ms to develop. We can then interpolate the Thelen data for females to find that they can develop 29.7 Nm in the same time. If we divide that moment by the corresponding female foot lever arm of 0.129 m, we obtain a foot force of 230 N or 23.5 Kgf. Hence, a first estimate for the threshold starting block force value for females would be 23.5 Kgf, or 6% less than that of the 25 Kgf male value. However, this estimate fails to take into account the higher strengths [6] and the more rapid rate of force development of sprinters [7,8], so we next take these effects into account.

We used MATLAB (2011A, The Mathworks, Natick, MA) to fit a 8^th^-degree polynomial to the rapid plantarflexor torque development vs. time curves measured for healthy young male and female non-athletes by Thelen et al. [4]. Each curve was then normalized to its peak torque value. Young females in the original Thelen study initiated their onset of plantarflexor torque 16 ms prior to the young males, so we retained this female temporal advantage because of evidence that females detect auditory cues before males [9,10]. We multiplied the amplitude of the plantarflexor torque development curves by the mean peak values measured at 30 deg/s by elite male and female sprinters, namely 103 Nm and 85 Nm, respectively [6]. The plantarflexor torque-time relationships were then transformed into force-time relationships by dividing by the sex-specific foot lever arms used above. Next, we need to account for male sprinters developing a 25% more rapid rate of maximal force development in their plantarflexors than untrained young males [7], and females sprinters developing a 7% more rapid force than untrained young females [8]. Therefore, the Thelen et al. force-time relationships curves were differentiated to determine the peak force development value for healthy young male and female non-athletes. The time scale on the plantarflexor force vs. time curve was then iteratively reduced until the differential of the force-time curve resulted in a peak value that was 7% and 25% greater for the female and male curves, respectively. This resulted in the prediction of a 7% and 20% compression in the time scale of the non-sprinter force-time relationships for females and males, respectively. We then found that female sprinters will generate 19.4 Kgf on a start block in the same time that male sprinters can develop the 25 Kgf, a 22% difference.

These estimates of a 6 to 22% lower threshold on the start blocks being needed for women are conservative because of the use of isokinetic strength data from the sprinters and a number of simplifying assumptions. These estimates could be refined through the use of experimental data obtained from sprinters actually competing at the Olympics. If the force threshold used at Beijing was higher than 25 Kgf, then the percentage difference we calculate would increase, and vice versa. Regardless, a lower force threshold for women would seem indicated.

**SUPPLEMENTAL REFERENCES**

1. Zemper ED. (2009) USA Track & Field Starters. USA Track & Field website. Available: <http://www.usatf.org/groups/officials/files/resources/track-events/starters-monograph-2009.pdf>. Accessed 2011 Sept 23.
2. Pain MT, Hibbs A (2007) Sprint starts and the minimum auditory reaction time. J Sports Sci. 25: 79–86.
3. Komi PV, Ishikawa M, Salmi J (2009) IAAF sprint start research project: Is the 100ms limit still valid? IAAF New Studies in Athletics. 24: 37-47.
4. Thelen DG, Schultz AB, Alexander NB, Ashton-Miller JA (1996) Effects of age on rapid ankle torque development. J Gerontol. 51A: M226-M232.
5. Wunderlich RE, Cavanagh PR (2001) Gender differences in adult foot shape: implications for shoe design. Med Sci Sport Exerc. 33: 605-611.
6. Alexander, MJL (1989) The relationship between muscle strength and sprint kinematics in elite sprinters. Can J Sport Sci. 14: 148-157.
7. Pääsuke M., Ereline J., Gapeyeva H (1999) Twitch contractile properties of plantar flexor muscles in power and endurance trained athletes. Eur J Appl Physiol. 80: 448–451.
8. Pääsuke M., Ereline J., Gapeyeva H., Torop T. (2002) Twitch contractile properties of plantarflexor muscles in female power-trained athletes. Med dello Sport. 55: 279–286.
9. Don M, Ponton CW, Eggermont JJ, Masuda A (1993) Gender differences in cochlear response time: an explanation for gender amplitude differences in the unmasked auditory brain-stem response. J Acoust Soc Am. 94: 2135-2148.
10. Trune DR, Mitchell C, Phillips, DS (1988) The relative importance of head size, gender and age on the auditory brainstem response. Hearing Res. 32: 165-174.
